# Both medial closing wedge and lateral opening wedge distal femoral osteotomy for valgus knee deformity can maintain leg length: A radiographic comparative study

**DOI:** 10.1002/jeo2.70184

**Published:** 2025-02-24

**Authors:** Shintaro Onishi, Youngji Kim, Oliver Adebayo, Hiroshi Nakayama, Christophe Jacquet, Matthieu Ollivier

**Affiliations:** ^1^ Institute for Locomotion, Aix‐Marseille University, APHM, CNRS, ISM, Sainte‐Marguerite Hospital Marseille France; ^2^ Department of Orthopedic Surgery Hyogo Medical University Nishinomiya Japan; ^3^ Department of Orthopaedics Juntendo University Tokyo Japan; ^4^ Department of Trauma and Orthopaedic Surgery Royal Free London NHS Foundation Trust London UK

**Keywords:** lateral opening wedge distal femoral osteotomy, leg length discrepancy, medial closed wedge distal femoral osteotomy, valgus knee osteoarthritis

## Abstract

**Purpose:**

To compare the radiological outcomes of medial closed wedge distal femoral osteotomy (MCWDFO) and lateral open wedge distal femoral osteotomy (LOWDFO), with a focus on evaluating leg length discrepancy (LLD). It was hypothesised that MCWDFO would result in a greater reduction in leg length compared to LOWDFO.

**Methods:**

Patients who underwent MCWDFO or LOWDFO for valgus deformity at a single institution between 2014 and 2022 with a minimum follow‐up of 1 year were included. Radiological assessment included hip–knee–ankle (HKA) angle, mechanical lateral distal femoral angle (mLDFA), medial proximal tibial angle (MPTA), length of the whole leg and femur and LLD. The difference between pre‐ and post‐operative values for each parameter is expressed as Δ. The radiological outcomes were statistically evaluated for each procedure.

**Results:**

Fifty‐two patients (26 MCWDFO and 26 LOWDFO) were included. No significant differences were observed between the two groups with respect to demographic data and radiological parameters such as HKA, mLDFA and MPTA. Although Δ length of the femur decreased post‐MCWDFO (−2.7 ± 0.6 mm) and increased post‐LOWDFO (+2.7 ± 0.4 mm), the Δ length of the whole leg post‐MCWDFO decreased (−0.5 ± 3.8 mm) and increased post‐LOWDFO (+1.7 ± 2.6 mm) (*p* < 0.001). The straight‐lengthening effect on the length of whole leg was significantly greater in MCWDFO than in LOWDFO (+2.0 ± 4.1 mm vs. −1.1 ± 2.5 mm, *p* > 0.001).

**Conclusions:**

The straight‐lengthening effect of alignment correction minimises changes in overall leg length, regardless of the specific DFO technique.

**Level of Evidence:**

Level III, retrospective comparative study.

**Abbreviations**
DFO: distal femoral osteotomy
HKA: hip–knee–ankle
HTO: high tibial osteotomy
Lf: length of femur
LLD: leg length discrepancy
LOWDFO: lateral open wedge distal femoral osteotomy
Lw: length of whole leg
MCWDFO: medial closed wedge distal femoral osteotomy
mLDFA: mechanical lateral distal femoral angle
mMPTA: mechanical medial proximal tibial angle
OA: osteoarthritis

## INTRODUCTION

Distal femoral osteotomy (DFO) is a well‐established treatment for lateral knee osteoarthritis (OA) with valgus deformity in the young active population [7, 13, 18, 20]. In general, DFOs for valgus malalignment can be performed using either medial closing wedge or lateral opening wedge osteotomy, and good clinical outcomes have been reported after using both techniques [20]. Leg length discrepancy (LLD) is a recognised effect following alignment corrective surgeries in the lower limb. Previous reports demonstrated that significant LLD can cause postural and gait abnormalities and low back pain [3, 8, 16, 19]. Therefore, it is imperative to consider the perioperative changes in leg length in the setting of preoperative planning in osteotomies around the knee. Previous studies have investigated LLD after high tibial osteotomy (HTO) [2, 9, 11, 21]. Generally, the leg length increased after medial opening wedge HTO, whereas the change in leg length after lateral closing wedge HTO was minimal despite the decrease in the bony length. On the other hand, there is limited data on LLD following DFO for symptomatic valgus knee deformity. Furthermore, no study has compared the change in leg length between medial closed wedge DFO (MCWDFO) and lateral opening wedge DFO (LOWDFO). Therefore, the aim of this study was to compare the radiological outcomes, specifically the perioperative changes in leg length, between MCWDFO and LOWDFO. It was hypothesised that MCWDFO would result in a greater reduction in leg length compared to LOWDFO.

## METHODS

### Study design and population

**Figure 1:**
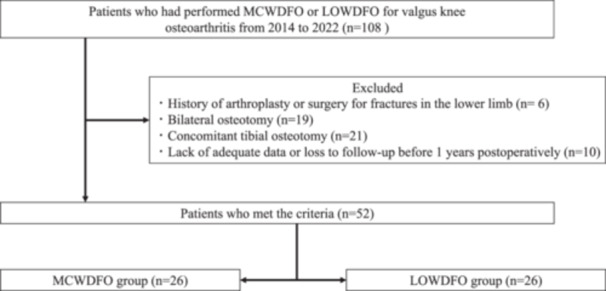
Flowchart of the patient selection process. LOWDFO, lateral opening wedge distal femoral osteotomy; MCWDFO, medial closed wedge distal femoral osteotomy.

This retrospective study enroled 108 consecutive patients with valgus knee OA who underwent MCWDFO or LOWDFO at a single, tertiary referral centre from 2014 to 2022. The inclusion criteria were symptomatic lateral compartmental knee OA with valgus malalignment. Exclusion criteria included prior history of arthroplasty or surgery for fracture in the lower limb, bilateral osteotomy during the study period, concomitant tibial osteotomy, and inadequate data or loss to follow‐up before one year post‐operatively. The process of patient selection is shown in Figure 1. The present study was approved by the Institutional Review Board of our institution (PADS24‐171b‐dgr), and written informed consent was obtained from all the patients.

### Surgical options and procedures

**Figure 2:**
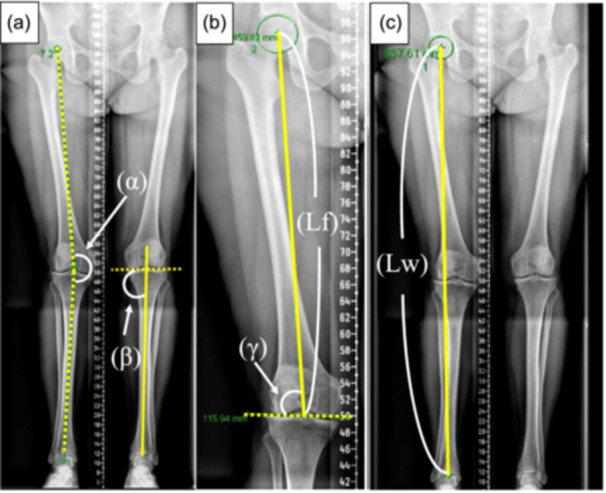
Measurement of radiological parameters using long leg radiograph (a–c). (α) Hip–knee–ankle angle. (β) Mechanical medial proximal tibial angle. (γ) Mechanical lateral distal femoral angle. (Lf) Length of the femur is defined as the distance between the centre of the femoral head and distal femoral joint surface. (Lw) Length of the whole limb is defined as the distance between the centre of the femoral head and the centre of tibial plafond.

Surgical decisions were based on radiological review of weight‐bearing long‐leg radiographs, assessing the hip–knee–ankle (HKA) angle, mechanical medial proximal tibial angle (mMPTA) and mechanical lateral distal femoral angle (mLDFA). (Figure 2a,b). Isolated DFO was indicated for symptomatic valgus knees with an isolated femoral deformity (mLDFA < 85°) without tibial extra‐articular deformity. The post‐operative HKA goal was 180° in all cases regardless of the arthritic grade, with the intended correction angle calculated using the reversed Miniaci method [4, 14]. Post‐operative mLDFA was planned as these values are within the normal range (85–90°) after the osteotomy. Taking into account the preoperative LLD, the choice between MCWDFO and LOWDFO was surgeon‐dependent.

Procedures were performed under general or spinal anaesthesia by a senior surgeon (MO). Arthroscopic examination and procedures for intra‐articular pathologies were performed as needed. The specific DFO technique (MCWDFO or LOWDFO) was executed as previously described [1, 17], utilising a minimally invasive sub‐vastus approach and an Activmotion® DFO plate (Newclip Technics). Intraoperative correction was based on the preoperatively measured distance corresponding to the correction angle. Allogenic bone graft was used to fill the osteotomy gap in LOWDFO cases.

### Post‐operative rehabilitation

All patients followed a standardised post‐operative protocol, including low molecular weight heparin for 1 month for thromboprophylaxis. The period of non‐weight‐bearing varied according to the bony healing status. In general, partial weight bearing was initiated between 4 and 6 weeks. Full weight‐bearing was allowed after the osteotomy hinge site was healed, typically between 6 and 8 weeks. Return to sports activities was recommended after 6 months, contingent upon adequate muscle strength and solid bony healing.

### Radiographic measurements

Pre‐ and post‐operative radiographic and clinical data were collected. Radiographic parameters included HKA, mMPTA, mLDFA, the length of the whole leg (Lw), the length of the femur (Lf) and LLD using the measurement tools within the Centricity Universal Viewer Zero Footprint (General Electric Healthcare) imaging software. Radiographs were considered adequate to measure if the patella was located in the centre of the knee with the knee in its fully extended position. The post‐operative radiographic measurement was conducted at least 1 year after the surgery. The Lw and Lf were defined as the distance between the centre of the femoral head and the centre of tibial plafond, and between the centre of the femoral head and the centre of distal femoral joint surface, respectively. All radiographic parameters including Lf and Lw were measured from whole‐leg radiographs (Figure 2b,c) with intended correction measured preoperatively. The LLD was calculated by the subtracting the Lw on the unaffected side from that of the affected side. The difference between the pre‐ and post‐operative values for each radiological parameter is expressed as delta (Δ). The wedge height based on assumed post‐operative LLD was calculated from the sum of the preoperative LLD and the intended correction gap distance. The femoral‐based assumed post‐operative LLD was defined as the sum of the preoperative LLD and the Δ Lf. The ‘straight‐lengthening effect’ on the Lw was calculated by subtracting the post‐operative femoral‐based assumed LLD from the post‐operative LLD to estimate the influence of alignment correction from valgus malalignment to neutral alignment on the leg length. In addition, the straight‐lengthening effect per each degree of correction was calculated by dividing the straight‐lengthening effect on the Lw by Δ mLDFA.

### Statistical analysis

Statistical analyses were performed using SPSS™ 12.0 (IBM Corporation). The normality of data distribution was assessed using the Shapiro–Wilk test. Statistical comparisons between MCWDFO group and LOWDFO group were made using the paired or unpaired *t* test for parametric data, the Mann–Whitney *U* test for non‐parametric variables, and the chi‐squared test for categorical outcomes, with the significant level set at or below 0.05. All variables were statistically compared between the two groups. The accuracy of the measurements was assessed using the intraclass correlation coefficient for intra‐ and interobserver reliability. Two independent observers reviewed every preoperative and post‐operative radiograph set twice in a blinded fashion with a 3‐week measurement interval to assess the intraclass correlation coefficients.

To assess the statistical power of this study, a two‐tailed post hoc power analysis was conducted using G*Power (version 3.1.9.6; Franz Faul, Universität Kiel) to compare the post‐operative Δ Lf between the MCWDFO and LOWDFO groups. The results show that the total sample size of 26 in each group could achieve an adequate power of 1 − β of 0.99, with an *α* of 0.05.

## RESULTS

All intra‐ and inter‐observer reliability were >0.8, indicating excellent reliability.

The study included 52 patients, 26 in the MCWDFO group and 26 in the LOWDFO group. The groups did not differ significantly in terms of demographic characteristics, except for a longer follow‐up period in the LOWDFO group (Table 1). Similarly, no significant differences were found between the groups regarding preoperative bony geometry or correction‐related parameters (Table 2). The mean Lw was maintained from 81.5 ± 9.9 cm preoperatively to 81.4 ± 9.9 cm post‐operatively after MCWDFO (*p* = 0.538), whereas these values were slightly increased from 77.8 ± 6.7 cm preoperatively to 78.0 ± 6.8 cm after LOWDFO (*p* = 0.002). Although the Δ Lf decreased by 2.7 ± 0.6 mm post‐operatively in the MCWDFO group and increased by 2.7 ± 0.4 mm in the LOWDFO group, the Δ Lw decreased by 0.5 ± 3.8 mm in the MCWDFO group and increased by 1.7 ± 2.6 mm post‐operatively in the LOWDFO group (Table 3). Preoperatively, the involved limbs were slightly shorter than the uninvolved limbs in this population (−1.6 ± 2.7 mm, *p* = 0.924). Post‐operatively, LLD increased significantly after LOWDFO (*p* = 0.002) but decreased slightly after MCWDFO (p = 0.538). The actual changes in LLD were smaller than the predicted changes based on wedge height or femoral length changes alone (Table 4). The straight‐lengthening effect on Lw was significantly greater after MCWDFO compared to LOWDFO (2.0 ± 4.1 mm vs. −1.1 ± 2.5 mm, *p* < 0.001).

**Table 1:** Clinical characteristics.

	Overall (*n* = 52)	MCWDFO (*n* = 26)	LOWDFO (*n* = 26)	*p*
Age (years)	39.9 ± 10.5 (24–64)	39.1 ± 10.4 (25–61)	40.7 ± 10.8 (24–64)	0.587
Male/female	30/22	16/10	14/12	0.404
Height (cm)	171.2 ± 6.6 (161–181)	171.4 ± 6.9 (161–181)	170.9 ± 6.3 (161–181)	0.770
Follow‐up period (years)	2.5 ± 1.1 (1–5)	2.0 ± 1.1 (1–4)	3.0 ± 1.0 (2–5)	0.002

*Note*: Values are expressed as mean and standard deviations, with ranges in parentheses. Statistical comparison in each domain between the MCWDFO and LOWDFO.

Abbreviations: LOWDFO, lateral opening wedge distal femoral osteotomy; MCWDFO, medial closed wedge distal femoral osteotomy.

**Table 2:** Comparison of radiological and correction‐related parameters between the MCWDFO and LOWDFO groups.

	MCWDFO (*n* = 26)	LOWDFO (*n* = 26)	*p*
Preoperative HKA	189.1 ± 1.0	188.8 ± 0.8	0.210
Preoperative mMPTA	85.3 ± 2.1	85.0 ± 2.4	0.577
Preoperative mLDFA	79.8 ± 1.0	80.0 ± 0.7	0.327
Intended correction angle (°)	8.2 ± 1.0	8.0 ± 0.7	0.327
Intended correction distance (mm)	9.0 ± 1.5	8.6 ± 0.9	0.228
Post‐operative HKA	179.8 ± 1.2	179.7 ± 1.3	0.824
Post‐operative mLDFA	87.5 ± 0.9	87.8 ± 0.7	0.230

*Note*: Values are expressed as mean and standard deviations.

Abbreviations: HKA, hip–knee–ankle; LOWDFO, lateral opening wedge distal femoral osteotomy; MCWDFO, medial closed wedge distal femoral osteotomy; mLDFA, mechanical lateral distal femoral angle; mMPTA, mechanical medial proximal tibial angle.

**Table 3:** Comparison of pre‐ and post‐operative limb length parameters between the MCWDFO and LOWDFO groups.

	MCWDFO (*n* = 26)	LOWDFO (*n* = 26)	*p*
Length of the whole leg (cm)			
Preoperative	81.5 ± 9.9	77.8 ± 6.7	0.123
Post‐operative	81.4 ± 9.9	78.0 ± 6.6	0.144
Δ length of the whole leg (mm)	−0.5 ± 3.8	1.7 ± 2.6	**0.020**
*p* *	0.538	**0.002**	
Length of the femur (cm)			
Preoperative	47.1 ± 2.4	46.9 ± 2.2	0.713
Post‐operative	46.9 ± 2.4	47.2 ± 2.3	0.638
Δ length of the femur (mm)	−2.7 ± 0.6	2.7 ± 0.4	**<0.001**
*p* *	**<0.001**	**<0.001**	

*Note*: Values are expressed as mean and standard deviations. Δ means deviations between the pre‐ and post‐operative values. Bold values indicate statistically significant differences.

Abbreviations: LLD, limb length discrepancy; LOWDFO, lateral opening wedge distal femoral osteotomy; MCWDFO, medial closed wedge distal femoral osteotomy.

* Statistical comparison between the pre‐ and post‐operative parameters.

**Table 4:** Comparison of limb length parameters between the MCWDFO and OWDFO groups.

	MCWDFO (*n* = 26)	LOWDFO (*n* = 26)	*p*
LLD (mm)			
Preoperative	−1.7 ± 3.1	−1.4 ± 2.3	0.744
Post‐operative	−2.2 ± 2.1	0.2 ± 1.6	**<0.001**
Δ LLD (mm)	−0.5 ± 3.8	1.7 ± 2.6	**0.020**
*p* *	0.538	**0.002**	
Wedge height based assumed post‐operative LLD (mm)	−10.7 ± 3.4	7.1 ± 2.7	**<0.001**
Femoral‐based assumed post‐operative LLD (mm)	−4.3 ± 3.1	1.3 ± 2.4	**<0.001**
Straight‐lengthening on the length of whole leg (mm)	2.0 ± 4.1	−1.1 ± 2.5	**<0.001**
Straight‐lengthening per each degree of correction (mm/degree)	0.3 ± 0.5	−0.1 ± 0.3	**<0.001**

*Note*: Values are expressed as mean and standard deviations. Δ means deviations between the pre‐ and post‐operative values. Bold values indicate statistically significant differences.

Abbreviations: LLD, limb length discrepancy; LOWDFO, lateral opening wedge distal femoral osteotomy; MCWDFO, medial closed wedge distal femoral osteotomy; mLDFA, mechanical lateral distal femoral angle.

* Statistical comparison between the pre‐ and post‐operative parameters. Wedge height based on the assumed post‐operative LLD was calculated from the sum of the preoperative LLD and the intended correction gap distance. Femoral‐based assumed post‐operative LLD was defined as the sum of the preoperative LLD and the Δ length of the femur. Straight‐lengthening effects on the length of whole leg were calculated by subtracting the femoral‐based assumed post‐operative LLD from the post‐operative LLD. Straight‐lengthening effect per each degree of correction was defined as the value of the straight‐lengthening effect divided by Δ mLDFA.

## DISCUSSION

The principal finding of this study is that both MCWDFO and LOWDFO for valgus knee deformity effectively maintain overall leg length and minimise LLD, despite the inherent differences in how each procedure affects femoral length. The straight‐lengthening effect associated with alignment correction appears to play a key role in mitigating changes in overall leg length, even though the Δ Lw and Δ LLD decreased significantly after MCWDFO compared to LOWDFO. The hypothesis that MCWDFO would lead to a greater reduction in leg length compared to LOWDFO was thus confirmed. However, both procedures successfully preserved the patient's original leg length with minimal change of LLD.

Only two studies have investigated the change in leg length change after LOWDFO [10, 12], with the results of their studies being contradictory. Kolb et al. [10] investigated the perioperative change in LLD using the same method as in our study. The mean value of the change in LLD was +7.9 mm, corresponding to a 7.4° correction of the mLDFA. In contrast, Madelaine et al. [12] showed that there were no significant changes in LLD before and after LOWDFO. However, the detailed methods of measuring the length of the whole leg and LLD were not described in their paper. The present study demonstrated that the Lw was slight but significantly increased by +1.7 mm when the mean correction angle in mLDFA was 7.8° after LOWDFO, even though the Lf increased by +2.7 mm. Of note, the mean preoperative LLD in the studies by Kolb et al. and Madelaine et al. was −6.4 and −7 mm, respectively. These values are higher than our values of −1.7 mm in the MCWDFO group and −1.4 mm in the LOWDFO group. A possible explanation for the difference between these studies could be the method of assessment, the degree of pre‐ and post‐operative flexion contracture, and the patient's medical history, such as the proportion of the bilateral valgus deformity or unilateral deformity due to previous trauma, surgical episode or other joint arthritis.

**Figure 3:**
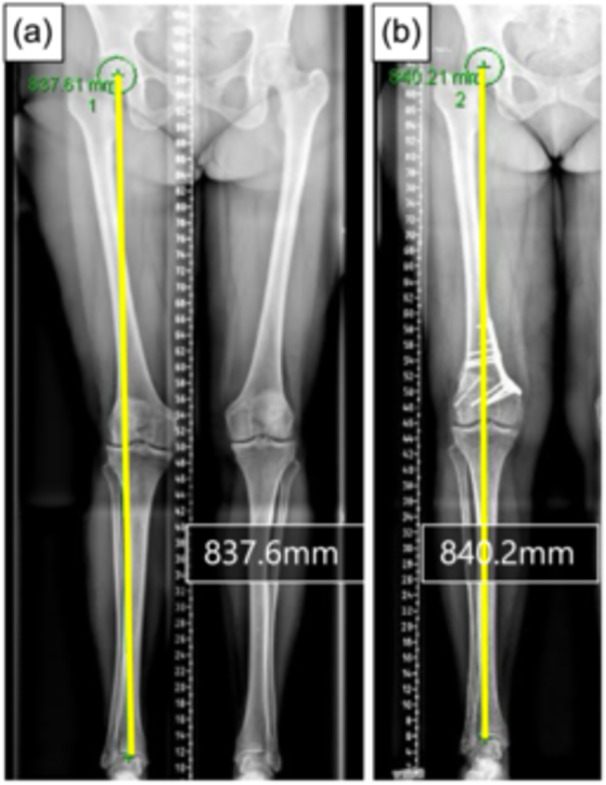
Representative cases who underwent MCWDFO. (a) Preoperative standing whole leg radiograph showing a valgus deformity of the right knee with a whole leg length of 837.6 mm. (b) Although the length of femur was decreased post‐operatively due to MCWDFO, the length of whole leg was slightly increased to 840.2 mm due to the straightening effect of alignment correction. MCWDFO, medial closed wedge distal femoral osteotomy.

Although there are few reports dealing with the leg length change after lateral closed wedge DFO concomitant with HTO for varus knee deformity [6, 15], no study has investigated the change in LLD after MCWDFO for valgus knee deformity. As mentioned above, the present study demonstrated that the preoperative Lw was generally shorter on the affected side than on the unaffected side. Previous studies have shown that the LLD of >5 mm is associated with an increased risk of hip and knee OA [5], and the LLD of >6 mm is associated with low back pain [16]. According to those previous findings, further shortening or excessive lengthening of the whole leg length after osteotomy is undesirable. Although MCWDFO and LOWDFO affected femoral length in opposite directions, the changes in overall leg length were less pronounced due to the straight‐lengthening effect of alignment correction. The straight‐lengthening effect can be due to the soft tissue lengthening that occurs, such as improvement in flexion contracture, tightening of the lateral tissues and widening of the lateral joint space due to the alignment correction. In addition, while most patients with a valgus knee deformity have radiographic hip adduction, these radiographic findings have improved after surgery (Figure 3). These changes in the position and direction of the femur may also contribute to the magnitude of the straight‐lengthening effect. The resulting Δ LLD values remained within acceptable limits, suggesting that both procedures can prevent problematic post‐operative LLD.

This study has several limitations, including its retrospective design, small sample size and the exclusion of potential confounding factors such as flexion contracture with a range of motion information, hinge fracture, intra‐articular findings such as meniscus or cartilage status and arthritic grade. Additionally, the clinical significance of the observed LLD changes was not evaluated. Despite these limitations, this study provides valuable insights, being the only study that has compared the radiological outcomes focusing on perioperative leg length changes and LLD between the MCWDFO and LOWDFO. Further prospective studies with larger sample sizes are needed to determine the optimal strategies to avoid unintended LLD in the setting of DFO for patients with valgus knee OA.

## CONCLUSIONS

Both MCWDFO and LOWDFO effectively maintain overall leg length in patients with valgus knee deformity, primarily due to the straight‐lengthening effect of alignment correction. While femoral length changes differ between the two procedures, the impact on overall leg length and LLD is minimal. These findings support using both MCWDFO and LOWDFO as viable options for managing valgus knee deformity while maintaining the leg length.

## AUTHOR CONTRIBUTIONS

All authors have contributed to the design, content and writing of the manuscript. All authors read and approved the final manuscript.

## CONFLICT OF INTEREST STATEMENT

Matthieu Ollivier has received consulting fees from Newclip Technics, Arthrex and Stryker. The remaining authors declare no conflicts of interest.

## ETHICS STATEMENT

Ethical approval for this study was obtained from the Institutional Review Board in our institution. Informed consent was obtained from all individual participants included in the study.

## Data Availability

The data that support the findings of this study are available from the corresponding author upon reasonable request.
